# The Dynamics of Plant Cell-Wall Polysaccharide Decomposition in Leaf-Cutting Ant Fungus Gardens

**DOI:** 10.1371/journal.pone.0017506

**Published:** 2011-03-10

**Authors:** Isabel E. Moller, Henrik H. De Fine Licht, Jesper Harholt, William G. T. Willats, Jacobus J. Boomsma

**Affiliations:** 1 Copenhagen Biocenter, Department of Biology, University of Copenhagen, Copenhagen, Denmark; 2 Centre for Social Evolution, Department of Biology, University of Copenhagen, Copenhagen, Denmark; 3 VKR Research Centre Pro-Active Plants, Section for Plant Glycobiology, Department of Plant Biology and Biochemistry, Faculty of Life Sciences, University of Copenhagen, Frederiksberg, Denmark; Centre National de la Recherche Scientifique, France

## Abstract

The degradation of live plant biomass in fungus gardens of leaf-cutting ants is poorly characterised but fundamental for understanding the mutual advantages and efficiency of this obligate nutritional symbiosis. Controversies about the extent to which the garden-symbiont *Leucocoprinus gongylophorus* degrades cellulose have hampered our understanding of the selection forces that induced large scale herbivory and of the ensuing ecological footprint of these ants. Here we use a recently established technique, based on polysaccharide microarrays probed with antibodies and carbohydrate binding modules, to map the occurrence of cell wall polymers in consecutive sections of the fungus garden of the leaf-cutting ant *Acromyrmex echinatior*. We show that pectin, xyloglucan and some xylan epitopes are degraded, whereas more highly substituted xylan and cellulose epitopes remain as residuals in the waste material that the ants remove from their fungus garden. These results demonstrate that biomass entering leaf-cutting ant fungus gardens is only partially utilized and explain why disproportionally large amounts of plant material are needed to sustain colony growth. They also explain why substantial communities of microbial and invertebrate symbionts have evolved associations with the dump material from leaf-cutting ant nests, to exploit decomposition niches that the ant garden-fungus does not utilize. Our approach thus provides detailed insight into the nutritional benefits and shortcomings associated with fungus-farming in ants.

## Introduction

Leaf-cutting ants are conspicuous New-World herbivores comprised of ca. 45 species divided into two genera, *Atta* and *Acromyrmex*. They form the monophyletic crown group of the attine fungus-growing ants, capable of harvesting over half a ton per year of live vegetation per colony and playing a significant role in nutrient transport and (sub)tropical ecosystems [1], [2].

The plant cell wall is composed of structurally complex polysaccharides which are compactly arranged and extremely resistant to degradation [3]. In order to deconstruct plant biomass into usable nutrients such as simpler sugars, leaf-cutting ants rely on an obligate symbiosis with fungus gardens that are assemblies of microbes dominated by the basidiomycete fungus *Leucocoprinus gongylophorus* (Agaricales: Agaricaceae) [1], [4]. While leaf-cutting ants provide their fungus gardens with plant material to sustain its growth, the fungus provides food for the ants and their brood in the form of specialized inflated hyphal tips (gongylidia) which the ants excise, consume and feed to their larvae [5], [6]. Fungus gardens are maintained in underground nest chambers where worker ants provide a clean environment for garden growth and express multiple hygienic behaviours to inhibit parasitic fungi and other unwanted microorganisms, usually assisted by a combination of aseptic glandular secretions and symbiotic bacteria producing antibiotics [1], [7]. The unique characteristics of these multipartite ant-symbiont relationships have led this mutualism to become a model system for studying social evolution at multiple levels [8]–[11].

*Atta* and *Acromyrmex* workers deposit small leaf fragments in the upper and outer-most regions of the fungus garden, which are then progressively metabolized and transformed into fungal biomass in the middle and lower sections [1], [12]. This implies that different stages of plant degradation are accomplished in consecutive sections of the garden, which is to some extent reflected in their visual appearance: a dark colored top layer with newly incorporated leaf material, a middle layer where the fungal biomass increases substantially and where clusters of gongylidia are most abundant [12], and a bottom layer with dense mycelial biomass and the remaining non-degraded plant substrate. Exhausted fungus garden material is continuously removed from the lowest sections by the ant workers and deposited in debris piles away from the fungus garden [13], [14].

The ability of fungus gardens to efficiently degrade and metabolise fresh leaf material may explain why *Atta* leaf-cutting ants in particular have become such complex and highly evolved animal societies with colonies of up to five million workers and extensive division of labour among worker castes [6], [15]. However, the precise mechanisms and sequence of degradation events in fungus gardens remain obscure. Relative proportions of plant substrates in consecutive garden sections are little understood and we have no knowledge about the extent to which plant cell wall properties affect the ants' selection criteria for accepting plant substrates into the garden, and for discarding old garden material with unused substrate. Without such information it is impossible to fully understand the dynamic processes that underpin plant biomass conversion in this symbiosis, and the resulting ecological footprint of these agricultural pest ants, which cause billions of dollars worth of damage each year [1].

Previous studies have utilised information about enzyme activities to infer aspects of substrate degradation both in naturally maintained fungus gardens [16]–[19] and in symbiont cultures grown *in vitro* [20]–[24]. These studies suggest that enzyme activities originate primarily from the symbiotic fungus, but that yeasts and bacteria residing in the fungus garden may also contribute [25]–[27]. Taken together, they indicated that *L. gongylophorus* mainly degrades proteins, starch, and plant cell wall polysaccharide components such as pectins and cross-linking glycans (also known as ‘hemicelluloses’), whereas cellulose remains largely intact. However, this indirect evidence remains controversial [15], [21], [22], [28]–[30], because most enzyme assays used single or very few highly specific substrates at any one time, which is problematic because the degradation of individual plant cell wall polysaccharides often requires the simultaneous action of complex multi-enzyme systems [31], [32].

**Figure 1:**
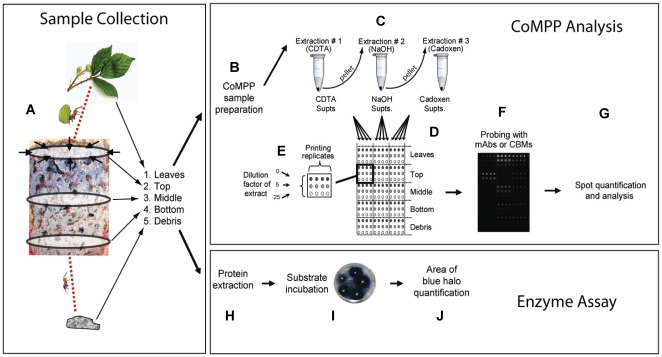
Analysis of plant degradation in leaf-cutting ant fungus gardens. Comprehensive Microarray Polymer Profiling (CoMPP) (A–G) and enzymatic assays (H–J) were used to assess plant degradation in *Acromyrmex echinatior* fungus gardens. (A) Leaf-cutting ants collect and transport fragments of fresh leaves back to the fungus garden where they are further fragmented and deposited in the top layer. The gradual degradation of cell wall polysaccharides, as the fungus garden grows upwards into the new substrate material, results in the plant material moving downwards as it is degraded because debris consisting of old fungus and exhausted substrate material is removed from the bottom of the fungus garden and discarded by the ants. The main steps in our CoMPP technique were: (A) collection of replicate material from leaves, top, middle and bottom layers of fungus gardens, and debris; (B) sample preparation by homogenization and precipitation of cell wall polymers; (C) sequential extraction of cell wall components with CDTA, NaOH and cadoxen; (D) printing of polysaccharides as microarrays using a robot, three concentrations, and four replicates (E); (F) probing of microarrays with monoclonal antibodies (mAbs) or carbohydrate binding modules (CBMs); (G) spot quantification and analysis. The activity of enzymes in the corresponding samples was approximated with azurine dyed and cross-linked (AZCL) polysaccharides substrates: (H) Protein extraction in tris buffer, (I) Substrate incubation, and (K) quantification of the area of blue halo (see **Text S1** for detailed methods).

A recently established technique, comprehensive microarray polymer profiling (CoMPP), utilizes carbohydrate microarray-based technology to obtain detailed information about the relative abundance of numerous plant cell wall polysaccharides within a set of biological samples [33]–[37]. This technology is underpinned by the availability of a large number of monoclonal antibodies (mAbs) and carbohydrate binding modules (CBMs) with specificities for defined glycan structures (epitopes) occurring on plant cell wall polysaccharides (**Table S1**). CoMPP does not provide information about the absolute levels of polysaccharides, but compared to conventional techniques for cell wall analysis such as monosaccharide composition analysis, CoMPP has the advantage that it provides information about glycan epitopes that can be assigned with confidence to specific polysaccharides. The technique is well suited for tracking detailed changes in cell wall components in complex biological systems and particularly so when a paucity of prior information about cell wall composition complicates the interpretation of data from conventional biochemical techniques [35], [36], [38]. We therefore used CoMPP to map the distribution of polysaccharide epitopes within consecutive sections of the fungus garden of *Acromyrmex echinatior* leaf-cutting ants, as well as in the leaves provided as forage and the debris discarded by the ants (**Figure 1**). Our findings reveal that fungus gardens only partially degrade the plant material provided by the ants and leave the cellulose-rich components largely untouched.

## Materials and Methods

Four queenright colonies of the leaf-cutting ant *A. echinatior* were excavated in Gamboa, Panama, in May 2003 (#Ae220) and May 2007 (#Ae332, #Ae334, #Ae356). The colonies were transported to the University of Copenhagen, Denmark, where they were maintained in a climate controlled room at 25°C, 70% RH, on a diet of bramble leaves (*Rubus* spec), dry rice and pieces of fruit. Three months prior to sampling, the colonies were supplied with bramble-leaves only, to make sure that fungus gardens were exclusively handling fresh leaf material by the time of sampling. Bramble leaves are highly suitable as forage as they allow *Acromyrmex* lab colonies to grow to their natural size and produce winged sexuals periodically. Five different categories of samples were collected from the four colonies in September 2008 (**Figure 1A**): (1) Fresh bramble leaves, (2) The newly established top section of the fungus garden, (3) The middle section of the fungus garden containing gongylidia, (4) The bottom section of the fungus garden (see also description in [12]), and (5) The debris pile consisting of waste material removed from the fungus garden by the ants. Samples in each category were collected in eight replicate (ca. 5 g fresh weight each), pooled, and stored at -20°C until further use.

### Comprehensive microarray polymer profiling (CoMPP)

An overview of the procedure used for CoMPP analysis is shown in **Figure** **1B–G**. Briefly, samples were homogenised and cell wall polysaccharides sequentially extracted from 10 mg dry weight of each sample as previously described [33] (**Figure 1B,C**). Supernatants containing extracted cell wall polymers were spotted as microarrays onto nitrocellulose membrane (Schleicher and Schuell, Dassel, Germany) (**Figure 1D**) and each sample was represented on arrays in three concentrations (the original extraction plus two five-fold dilutions) and as four printing replicates (a total of twelve spots per sample) (**Figure 1E**). Printing was carried out using a microarray robot (Microgrid II, Genomic Solutions, Cambridge, UK) equipped with split pins (Microspot 2500, Genomic solutions, Cambridge, UK). The arrays were probed with mAbs and CBMs and developed as described previously [33] (**Figure 1F,G**). Details of the mAbs or CBMs used to probe the arrays are listed in **Table S1.** All probes were obtained from PlantProbes (Leeds, UK) except BS 400-2 that was obtained from BioSupplies (Melbourne, Australia).

**Figure 2:**
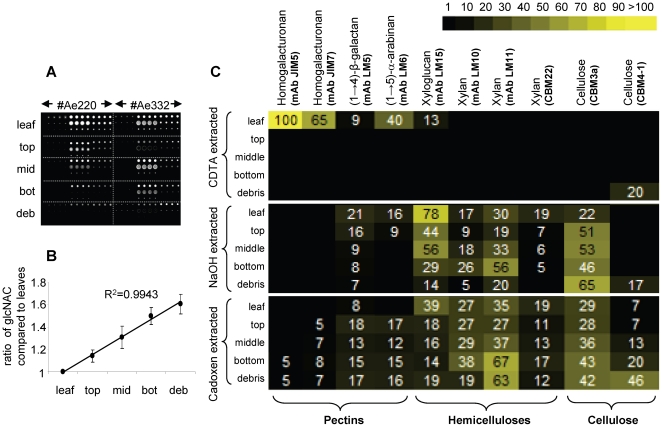
Comprehensive microarray polymer profiling (CoMPP) of *Acromyrmex echinatior* fungus gardens. (A) An example of a CoMPP microarray populated with material from two colonies and probed with the anti-xyloglucan mAb LM15. (B) Change in average fungal biomass during the processing of leaf material in *A. echinatior* fungus gardens (±SE). Fungal biomass was determined by measuring the amount of chitin present in fungal cell walls from five different sections: leaves, top, middle and bottom of the fungus garden, and debris pile from the four replicate colonies. (C) The distribution of cell-wall polysaccharides in fungus gardens represented as a heatmap where mean CoMPP spot signals (numbered values, corrected for changes in fungal biomass) are correlated to colour intensity. Sample locations and extraction conditions are shown on the left, polysaccharide epitopes and corresponding monoclonal antibodies mAbs and carbohydrate binding modules (CBM), are shown on the top. The highest corrected mean signal value in the data set was set to 100 and all other values adjusted accordingly. All data are averages of four experiments.

Spot signals were quantified and analysed using Imagene 6.0 microarray analysis software (Biodiscovery, http:/www.biodiscovery.com) as previously described [33] (**Figure 2A**). The variation in degradation patterns across colonies was analyzed with a general linear mixed model after correcting mean spot signal data for differences in fungal biomass (**Figure 2B**). Heatmaps (**Figure 2C**) were constructed from the mean spot signal data using online heatmapper software (http://bbc.botany.utoronto.ca/ntools/cgi-bin/ntools) (see **Text S1**).

### Fungal biomass and enzyme activity assays

Fungal biomass in each of the five sample locations (leaves, top, middle, bottom of the fungus garden, and debris) was corrected between samples by measuring N-acetylglucosamine (GlcNAC) the principal monosaccharide constituent of the polymer chitin, and a major component of fungal but not plant cell walls. The procedures used were slightly modified from previous studies [39], [40] and are described in detail in the **Text S1**.

AZCL-polysaccharide colorimetric gel-based assays (Megazyme, Bray, Ireland) were used to determine enzyme activity in the fungus garden as previously described [12], [19]. We applied our AZCL analysis to three sample locations within the fungus garden (top, middle, bottom) that were used in the the CoMPP analysis. See **Text S1** for details.

## Results

### Detailed mapping shows that individual plant cell wall polymers are differentially degraded

Our results provide detailed insight into the dynamic turnover of polysaccharide epitopes across the five sampling locations. The three major classes of cell wall polysaccharides, pectins, hemicelluloses and cellulose are held together in walls with increasing degrees of firmness by different chemical bonds and supra-molecular associations. These were sequentially extracted from the fungus garden using three solvents CDTA, NaOH and cadoxen, respectively, so that our results provide information not just about the relative abundance of polymers *per se*, but also about the disassembly of higher order cell wall architectures.

**Figure 3:**
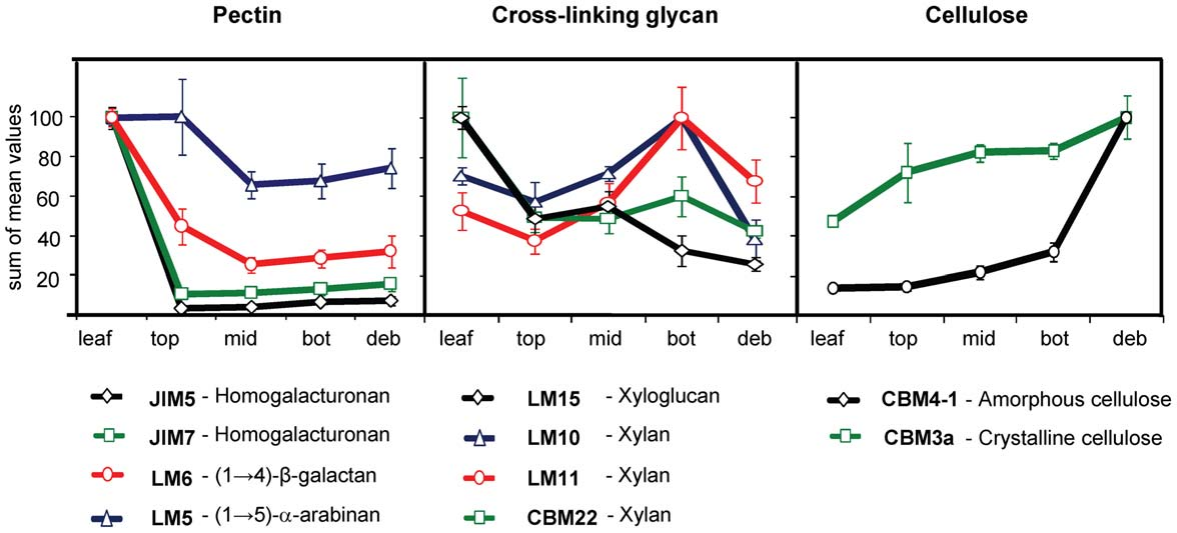
Overall changes in polysaccharide occurrence over five sampling locations. The relative amount of pectins, hemicelluloses and cellulose across five different sampling locations (leaves, top, middle, and bottom layers of the fungus garden, and debris) were determined by CoMPP analysis. Data are sums that combine the mean signals for all three extractions (CDTA, NaOH and Cadoxen), averaged across the four colonies and scaled relative to 100 for each polysaccharide epitope corresponding to a specific mAb or CBM. Error bars represent standard errors (±SE).

A heatmap showing the mean CoMPP signals obtained for all the mAbs and CBMs is shown in **Figure 2C**. Combining these data across the three extractions provided an overview of the changes in cell wall epitope levels as plant material is processed through the fungus garden (**Figure 3**) and of the overall fold changes in polysaccharides relative to levels in the starting leaf material (**Figure S1**). The average levels of fungal cell wall biomass increased relative to leaf weight by a factor of 1.14, 1.31, 1.5 and 1.6 in the top, middle, bottom and debris samples, respectively (**Figure 2B**). This regression, which reflects the progressive conversion of plant biomass into fungal biomass, was used to normalise the mean signal values from the CoMPP arrays. ANOVA revealed no significant variation in polysaccharide abundance between the four fungus gardens in our study, confirming that our experimental design produced consistently repeatable results (**Table S2**).

Homogalacturonan (HG) backbone domains of pectin (recognised by mAbs JIM5 and JIM7) were substantially degraded in, or even prior to incorporation in, the top layer of the fungus garden (**Figure 2C** and **Figure 3**). Of all the polysaccharide epitopes analysed in this study, HG was the substrate compound most significantly degraded in the fungus garden system (13 and 6.3 fold, respectively, for JIM5 and JIM7, **Figure S1**). Material extracted from leaves provided relatively high signals for both these anti-HG mAbs, but the same HG epitopes were essentially absent from all other sampling locations (**Figure 2C** and **Figure 3**). Both mAbs bind to partially methyl-esterified HG, but JIM5 binds preferentially to HG with a relatively low degree of methyl-esterification (DE) whilst JIM7 binds preferentially to HG with a relatively high DE. The decrease in JIM7 binding may have resulted partly from a reduction in methyl-ester groups on pectin polymers. This could have occurred as a result of endogenous pectin methyl-esterase activity in the leaves (possibly triggered by ant cutting), or by exposure to exogenous pectin methyl-esterases produced by microorganisms that the leaf material came into contact with during transport or storage, prior to incorporation into the fungus garden. However, a reduction in DE would not cause a reduction in JIM5 binding so our data indicate that cleavage of the HG backbone in pectin molecules also occurred to a significant extent. The (1→4)-β-D-galactan and (1→5)-α-L-arabinan side chains of pectin (recognised by mAbs LM6 and LM5, respectively) were detected only in the leaf material of CDTA-extracted samples, but signals persisted in NaOH and cadoxen extractions from other samples (**Figure 2C** and **Figure** **3**). Some HG epitopes were also present in cadoxen extracted samples. This indicates that some pectic domains were associated with hemicelluloses or cellulose, rendering them more recalcitrant to degradation [35].

Xyloglucan (XyG) (recognised by mAb LM15) and xylans (recognised by mAbs LM10, LM11 and CBM22) showed complex profiles of occurrence throughout the fungus garden system (**Figure 2C** and **Figure 3**). The relative level of the LM15 XyG epitope decreased sharply between leaves and samples from the top layer, increased slightly between top and middle layers and then decreased between middle layer and debris samples (**Figure 3**). Out of all hemicellulosic polysaccharides measured in this study, the LM15 XyG epitope decreased the most overall (3.8 fold) compared to levels in the starting leaf material (**Figure S1**). Changes in the overall relative levels of all three xylan-binding probes (LM10, LM11 and CBM22) were similar, with probe signals decreasing between leaves and samples from the top section, increasing between top and bottom sections (sharply in the case of LM10 and LM11), and then decreasing between bottom layer and debris samples (**Figure 3**). However, subtly different profiles were evident when the levels of xylan epitopes were compared across the different extraction treatments (**Figure 2C**). For both LM10 and LM11 the highest mean signals were obtained in bottom layer samples, but in contrast to LM10, LM11 epitope levels were also high in debris samples extracted with cadoxen. Levels of CBM22 epitope were highest in leaf material for both NaOH and cadoxen-extracted samples. For NaOH-extracted samples, levels then decreased across the other layers and the epitope was not detected in the debris. In contrast, CBM22 binding after cadoxen extraction persisted in debris samples (**Figure 2C**). Both LM10 and LM11 recognise unsubstituted (1→4)-β-D-xylans that are associated with secondary cell walls in dicotyledons, while LM11 also binds to more substituted xylans, such as arabinoxylan [41]. CBM22 also recognizes xylans with different degrees of substitution but has a distinctly different binding profile to LM11 on plant materials [42]. Our data therefore show that the degree to which xylan polymers are decorated with other sugars significantly affects their processing with the fungal garden.

The cellulosic epitopes recognised by CBM3a and CBM4-1 increased in relative abundance across the five sampling locations (**Figure 2C** and **Figure 3**). The levels of CBM3a and CBM4-1 epitopes were higher (2.1 and 7 fold, respectively) compared to the starting leaf material (**Figure S1**). This indicates that these epitopes were not significantly degraded and their relative abundance increased to commensurate with decreasing levels of other cell wall polymers. Whereas CBM3a is a type A CBM that binds to crystalline cellulose, CBM4-1 is a type B CBM that binds to internal amorphous regions of cellulose microfibrils [43]. Interestingly, whilst the level of CBM3a binding increased steadily throughout the fungal garden system, CBM4-1 binding was restricted mostly to cadoxen-extracted debris material. Cadoxen treatment is known to affect cellulose crystallinity and is thus likely to render it more amenable to CBM4-1 binding. However, the high level of CBM4-1 binding to debris is significant because it may reflect that the gradual removal of other cell wall polymers, particularly XyG, some of which coats cellulose microfibrils, alters the higher order structure of cellulose microfibrils [42].

### Enzyme activity often correlates with polysaccharide occurrence

Seven different azurine dyed and cross-linked (AZCL) polysaccharide substrates were used to assess the activities of cell wall degrading enzymes in top, middle, and bottom sections of fungus gardens (see **Table S3** for details). Enzyme activities were detected for the degradation of all seven polysaccharide substrates and in most cases, enzyme activities were correlated with the levels of cell wall epitopes, as monitored by CoMPP analysis (**Figure S2**). Exceptions to this were galactanase activity which was similar in all three zones, whilst galactan decreased from top to middle and remained at this level in the bottom section (**Figure S2A**), and xyloglucanase activity, which decreased from top to the middle section, whilst xyloglucan abundance was highest in the middle section of the fungus garden (**Figure S2C**). This indicates that enzyme activity generally reflects substrate availability and the positive rather than negative correlation between substrate and enzyme activities suggests an excess of substrate. However, the fact that galactan levels decreased whilst galactanase activity remained unchanged may suggest that galactan was not in excess and that a substantial proportion of this polymer was degraded. Although these findings provide useful insights into the mechanism of polysaccharide degradation in fungal gardens it should be noted that enzyme and CoMPP data cannot be precisely integrated because of the occurrence of multiple isoforms of many cell wall components. For example, variant XyGs exist with many different side chain configurations that can affect both antibody binding and enzyme activity.

## Discussion

### Pectin degradation starts almost immediately

One of our most striking findings was that pectin, particularly homogalacturonan, was extensively degraded in the time between when leaves are collected by the ants and incorporated into the top of the fungus garden, or in the top section itself. Leaf cutting ants first chew plant material into a pulp-like mass that is deposited in the fungus garden, after which the ants place droplets of fecal fluid containing polysaccharide degrading enzymes directly on top of the new substrate [44]. These enzymes include many pectinases (lyases and esterases), proteases and cellulases, which have been shown to originate from the fungal symbiont itself, passing unharmed through the ant gut and remaining active after deposition [44]. This mechanism potentially achieves a very rapid degradation of cell wall polymers and likely explains the almost complete absence of certain pectic polymers in the top section of the fungus garden. Our results are thus consistent with previous studies indicating that fecal fluid manuring is a key characteristic of this symbiosis because it accelerates access of the fungal hyphae to primary resources inside the plant cells [12], [18], [19], [24].

**Figure 4:**
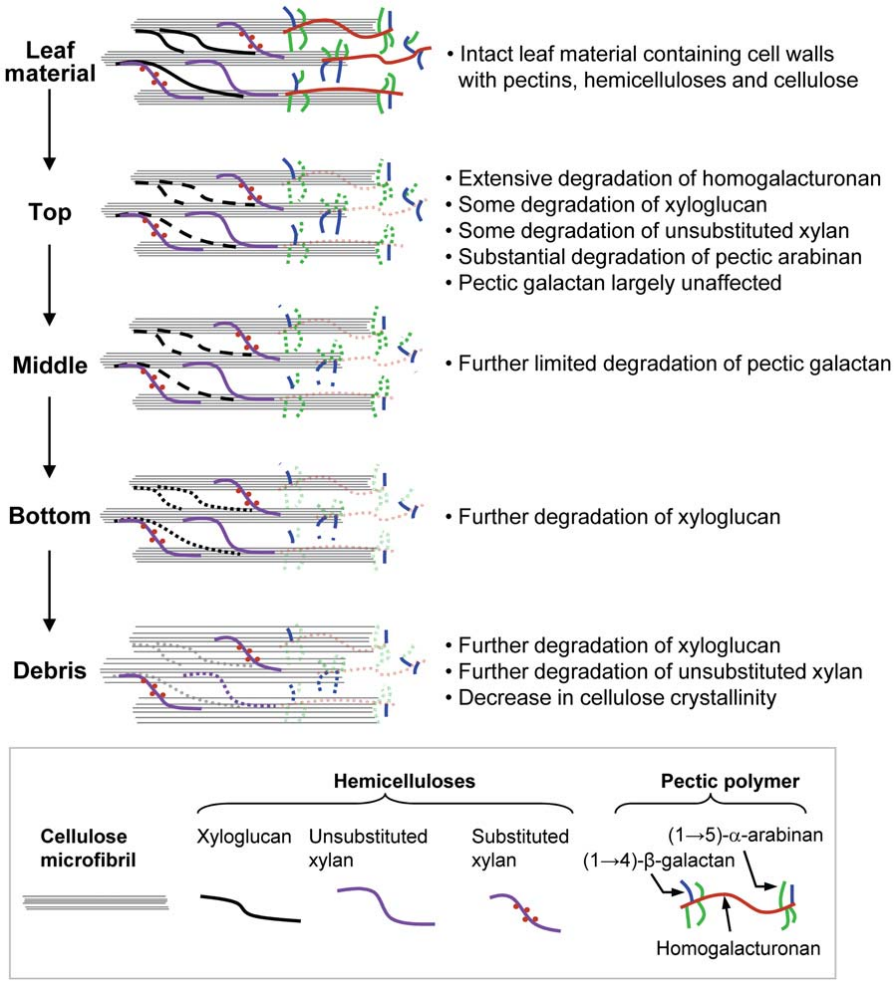
A simplified model of polysaccharide degradation in *Acromyrmex echinatior* fungus gardens. The diagram is a synthesis of the CoMPP results obtained in our study. It illustrates three major classes of polysaccharides (pectins, hemicelluloses and cellulose) that are typically present in leaves and the fate of individual polysaccharides when they are processed by the fungus gardens.

Recent studies using combinations of cell wall enzymes and antibodies have revealed that hemicelluloses are extensively masked by pectins, rendering them inaccessible to antibodies and therefore most likely enzymes as well [45]. These findings are relevant to the present study because they indicate that the extensive degradation of the pectic network that occurs prior to or just after deposition of leaf material in the top zone of fungal gardens is an essential pre-treatment for the effective utilisation of hemicellulases in the main body of fungal gardens. Pectin is a major structural component of plant cell walls, forming a gel-like matrix that is particularly abundant at cell wall interfaces in the middle lamella region of leaves, where it regulates intercellular adhesion [46] (**Figure 4**). There is also abundant evidence that dissociation of the pectin-matrix induces changes in physiological properties of plant cells that makes them more susceptible to microbial attack [47], [48]. Taken together this strongly suggests that the main function of pectin degradation is to give the fungal hyphae access to superior resources such as proteins and starch inside the plant cells, rather than pectin being an important nutrient source in its own right [19], [20], [23].

### Fungus gardens do not prioritize degradation of recalcitrant polysaccharides

While our analysis confirmed that protein and starch are likely to be the prime targets of leaf decomposition in leaf-cutting ant fungus gardens [19], it also provided compelling evidence for cellulose and xylan not being decomposition priorities. In the fungal gardens proper there was a gradual relative reduction of xyloglucan and unsubstituted xylan epitopes from the top to bottom zones and some further degradation of pectin side chains. As shown in the general overview **Figure 4**, the discarded debris consisted largely of cellulose and xylan, indicating that the relative levels of these polymers necessarily increased as a proportion of the total biomass. The increase in binding of CBM4-1, which preferentially targets amorphous cellulose [43], probably indicates that as other cell wall polymers are progressively removed, cellulose microfibrils become increasingly exposed and susceptible to extraction by cadoxen.

Our results are in broad agreement with previous studies based on the measurement of enzyme activity in fungus gardens [12], [16]-[18], [24]. In addition, our study provides a more detailed account of the degradation dynamics in leaf-cutting ant fungus gardens via direct assessment of the abundance of plant biomass components throughout the system. The use of CBMs 3a and 4-1 that recognise distinct structural forms of cellulose provided direct and compelling evidence that cellulose is not utilised as a substrate in *Acromyrmex echinatior* fungus gardens to any significant degree.

In contrast, a recent study provides evidence that cellulose is significantly degraded in field-collected fungus gardens of the leaf-cutting ant *Atta colombica* possibly by γ-proteobacteria isolated from the microbiomes of fungus gardens [27]. However these results may not be directly comparable because the authors measure absolute cellulose amounts without correcting for differences in fungal biomass, in contrast to relative abundance in our study. It is also puzzling that relatively small decreases in pectin or hemicellulose polymers were observed [27], since the presence of these polymers would likely limit the accessibility of cellulose degrading enzymes to their substrate [45]. Therefore these results for *Atta colombica* do not provide evidence that the fungal symbiont can degrade cellulose to any significant degree, rather that the microbial community of fungus gardens has this ability.

This apparent division of labour in cellulose-decomposing ability between the fungal symbiont and other garden microbes may resolve the paradox of some earlier studies, which either find evidence of some cellulose decomposition in fungus gardens [25], [27], [49], or find this activity is insignificant [20], [21], [23], [24]. The actual outcome could well be reinforced by active interference by the farming ants that fastidiously manage the relocation of waste and exhausted fungus material from the garden. Thus being an important structuring force of the microbial communities in their gardens [50]. As our data indicate, the question of ability is largely irrelevant when it is not prioritized by the ants because they discard older fungus garden material before any significant amount of cellulose or xylan is decomposed. Based on the diversity of expressed enzyme activities that we measured, it is likely that the fungus gardens retained a high degree of plasticity to cater for many different types of resources when they are made available, as demonstrated by many saprotrophic fungi that facultatively produce extracellular carbohydrate degrading enzymes [51]. As we show here, ample provisioning with fresh leaves allows the ants to prioritize resources that are of higher value and easier to process, rather than cellulose and xylan. However, we cannot exclude that cellulases may have a more significant role under sub-optimal foraging conditions, as for example in dry seasons, when fresh leaf material is less readily available.

### Comparative fungus garden perspectives: Ecological footprints and evolutionary opportunities

The approach developed in this study offers a versatile toolkit for studying evolutionary relationships between the degradation capacities of fungal symbionts of different genera of fungus-growing ants. There are more than 230 extant species of fungus-growing ants divided into 12 genera [9]. Only a single monophyletic group of fungus-growing ants, comprised of the genera *Atta* and *Acromyrmex,* has evolved actual fresh-leaf-cutting behaviour [1], [9], [15]. The majority of attine ants are less advanced and have much smaller colonies and fungus gardens the size of a tennis or table-tennis ball, which they provide with dead plant material and leaf litter [2], [9]. This suggests that the fungus gardens of these ant genera may utilize different suits of extracellular enzymes to facilitate the degradation and metabolism of plant cell wall polysaccharides [19]. This could imply that the profile of carbohydrate active enzymes in these gardens resembles what we here report for the lower sections of *A*. *echinatior* fungus. The clearly visible stratification of leaf-cutting ant fungus gardens is normally not observed in gardens of more basal attine ants, which may imply that this is a derived trait associated with the fairly recent [2] acquisition of a herbivorous niche with richer and more abundant resources. However, the advance into this foraging niche has apparently also made the utilization of substrate relatively wasteful, as residues appear to be discarded as soon as the pectins, starches and proteins typical for fresh leaves have been degraded. This hypothesis was recently formulated based on comparative AZCL enzyme activity data across fungus gardens of eight genera of attine ants [19], and appears consistent with the results provided here.

The fact that not all plant biomass is utilized, implies that much more plant substrate is needed to sustain leaf-cutting ant colonies than would be the case if all plant cell wall components were used [21], [22]. This may explain the large detrimental effect of leaf-cutting ant herbivory in (semi)natural and agricultural ecosystems [52], the elaborate and specialized waste management behaviours seen in *Atta* leaf-cutting ants [13], [14], and the large waste dumps that these colonies maintain. If wastefulness was an inevitable byproduct of acquiring the novel herbivorous niche, cellulose-rich dumps must have been an integral part of leaf-cutting ant fungus-farming since the *Atta* and *Acromyrmex* clade arose about ten million years ago [9]. This relatively long time span is consistent with distinct communities of microorganisms having evolved in these dumps [50], together with highly diverse communities of invertebrate commensals that directly or indirectly exploit the resources that the ants discard [1], [15], and with leaf-cutting ant colonies having major effects on local nutrient cycling [53], [54].

## Supporting Information

Table S1 **The binding specificities of monoclonal antibodies (mAb) and carbohydrate binding molecules (CBM) probes used in this study.**
(DOC)

Table S2 **Analysis of colony-level variation with a general linear mixed model.** The effect of colony-level variation on the distribution of cell wall polysaccharides was analysed for each mAb and CBM with a general linear mixed model (see text S1 for details). P-values are not corrected for multiple testing as this would render the test's too-conservative when colony variation is expected not to influence the results (Bonferroni correction increase the significance level to α  = 0.005). These results indicate that there was no significant variation in the occurrence of cell wall polysaccharides across the fungus gardens of the four colonies used in our study.
(DOC)

Table S3 **Substrates used to assess the activity of cell wall degrading enzymes in fungus gardens.**
(DOC)

Figure S1 **Fold changes (decreases or increases) in polysaccharide occurrence.** Numerical values represent the degree of change in polysaccharide occurrence between the leaf material input to the fungus garden and the debris output from the fungus garden. Individual mAbs and CBMs and their corresponding polysaccharide epitopes are listed in the figure. * indicate that fold-changes were significantly different from 1 (ANOVA, p < 0.05). NA indicates that statistical analysis could not be performed on CBM4-1 as this epitope was only available for analysis from two colonies. Error bars represent standard error (±SE).
(TIF)

Figure S2 **Enzyme activity in fungus gardens of** ***Acromyrmex echinatior*** **.** Bar graphs (A-H, left y-axis) indicate enzyme activity as halo area (cm^2^) in the three different sampling locations of the fungus garden as determined by AZCL-polysaccharide plate assays. Line graphs (A-H, right y-axis) indicate relative abundances of the corresponding polysaccharides as determined by CoMPP analysis (the same data as in Figure 3 but not adjusted to become a fraction of 100). Enzyme substrates and the corresponding mAbs or CBMs used in the analysis are indicated for each graph (see also Table S1). Enzyme data for (1-4)-β-D-xylan are shown twice (D and F) as both mAb LM10 and CBM22 detect this substrate. Error bars represent standard errors across four colonies measured.
(TIF)
