# Supplementary material for: The Dynamics of Plant Cell-Wall Polysaccharide Decomposition in Leaf-Cutting Ant Fungus Gardens
Source: PLoS One. 2011 Mar 10;6(3):e17506. doi: 10.1371/journal.pone.0017506 (PMC3053354; doi:10.1371/journal.pone.0017506)
Supplement: Table S1 — The binding specificities of monoclonal antibodies (mAb) and carbohydrate binding molecules (CBM) probes used in this study. (DOC) [file pone.0017506.s001.doc]

**Table S1**

| **Polysaccharide class** | **Probe** | **Specificity** | **References** |
| --- | --- | --- | --- |
| Pectin | JIM5 | partially methyl -esterified homogalacturonan* | [1] |
|  | JIM7 | partially methyl -esterified homogalacturonan** | [1] |
|  | LM5 | (1→4)-β-D-galactan | [2] |
|  | LM6 | (1→5)-α-L-arabinan | [3] |
| Cross-linking glycans | LM15 | xyloglucan | [4] |
|  | LM10 | (1→4)-β-D-xylan^+^ | [5] |
|  | LM11 | (1→4)-β-D-xylan^++^ | [5] |
|  | CBM22 | (1→4)-β-D-xylan^+++^ | [6] |
| Cellulose | CBM3a | cellulose, crystalline | [7] |
|  | CBM4-1 | cellulose, amorphous | [8] |

*binds preferentially to homogalacturonan with a low degree of methyl esterification

**binds preferentially to homogalacturonan with a low degree of methyl esterification

^+^binds to unsubstituted (1→4)-β-D-xylan

^++/+++^bind to unsubstituted and substituted (1→4)-β-D-xylan

see **Text S1** for references
