# Supplementary material for: The Dynamics of Plant Cell-Wall Polysaccharide Decomposition in Leaf-Cutting Ant Fungus Gardens
Source: PLoS One. 2011 Mar 10;6(3):e17506. doi: 10.1371/journal.pone.0017506 (PMC3053354; doi:10.1371/journal.pone.0017506)
Supplement: Table S2 — Analysis of colony-level variation with a general linear mixed model. The effect of colony-level variation on the distribution of cell wall polysaccharides was analysed for each mAb and CBM with a general linear mixed model (see text S1 for details). P-values are not corrected for multiple testing as this would render the test's too-conservative when colony variation is expected not to influence the results (Bonferroni correction increase the significance level to α = 0.005). These results indicate that there was no significant variation in the occurrence of cell wall polysaccharides across the fungus gardens of the four colonies used in our study. (DOC) [file pone.0017506.s002.doc]

**Table S2**

| **antibody** | |  | **JIM5** | **JIM7** | **LM5** | **LM6** | **LM10** | **LM11** | **LM15** | **CBM22** | **CBM3a** | **CBM4-1** |
| --- | --- | --- | --- | --- | --- | --- | --- | --- | --- | --- | --- | --- |
| sample location | F | | 199.48 | 62.95 | 23.26 | 64.20 | 9.44 | 9.12 | 55.57 | 13.21 | 0.79 | 3.50 |
|  | *p* | | <0.0001 | <0.0001 | <0.0001 | <0.0001 | 0.0011 | 0.0013 | <0.0001 | 0.0002 | 0.5512 | 0.0408 |
| extraction method | F | | 29.85 | 30.84 | 9.27 | 14.01 | 97.39 | 55.95 | 40.34 | 45.55 | 39.81 | 2.94 |
|  | *p* | | 0.0008 | 0.0007 | 0.0146 | 0.0055 | <0.0001 | 0.0001 | 0.0003 | 0.0002 | 0.0003 | 0.1290 |
| sample*extraction | F | | 145.46 | 51.51 | 15.23 | 45.65 | 3.20 | 11.49 | 5.87 | 5.58 | 4.03 | 1.78 |
|  | *p* | | <0.0001 | <0.0001 | <0.0001 | <0.0001 | 0.0129 | <0.0001 | 0.0003 | 0.0005 | 0.0037 | 0.1321 |
| colony | F | | 2.14 | 2.01 | 3.41 | 4.58 | 1.45 | 3.38 | 1.44 | 2.98 | 0.80 | 1.11 |
|  | *p* | | 0.2222 | 0.2471 | 0.0879 | 0.0593 | 0.2968 | 0.0885 | 0.3320 | 0.0846 | 0.5298 | <0.0001 |
| sample*colony | F | | 0.55 | 0.77 | 1.53 | 1.03 | 1.87 | 1.50 | 0.87 | 2.49 | 2.22 | 4.57 |
|  | *p* | | 0.8577 | 0.6734 | 0.1827 | 0.4580 | 0.0932 | 0.1911 | 0.9960 | 0.0274 | 0.0469 | 0.9990 |
