# Supplementary material for: The Dynamics of Plant Cell-Wall Polysaccharide Decomposition in Leaf-Cutting Ant Fungus Gardens
Source: PLoS One. 2011 Mar 10;6(3):e17506. doi: 10.1371/journal.pone.0017506 (PMC3053354; doi:10.1371/journal.pone.0017506)
Supplement: Table S3 — Substrates used to assess the activity of cell wall degrading enzymes in fungus gardens. (DOC) [file pone.0017506.s003.doc]

**Table S3**

| **polysaccharide class** | **substrate** | **enzyme activity** |
| --- | --- | --- |
| pectin | AZCL-galactan | galactanase |
|  | AZCL-debranched arabinan | arabinase |
| cross-linking glycans | AZCL- xyloglucan (tamarind) | xyloglucanase |
|  | AZCL- xylan (birch or oat) | xylanase |
|  | AZCL-arabinoxylan (wheat) | xylanase |
| cellulose | AZCL- hydroxyethyl cellulose | cellulase |
|  | AZCL- barley β-glucan | glucanase |
