# Supplementary figures and images for: The Dynamics of Plant Cell-Wall Polysaccharide Decomposition in Leaf-Cutting Ant Fungus Gardens
Source: PLoS One. 2011 Mar 10;6(3):e17506. doi: 10.1371/journal.pone.0017506 (PMC3053354; doi:10.1371/journal.pone.0017506)

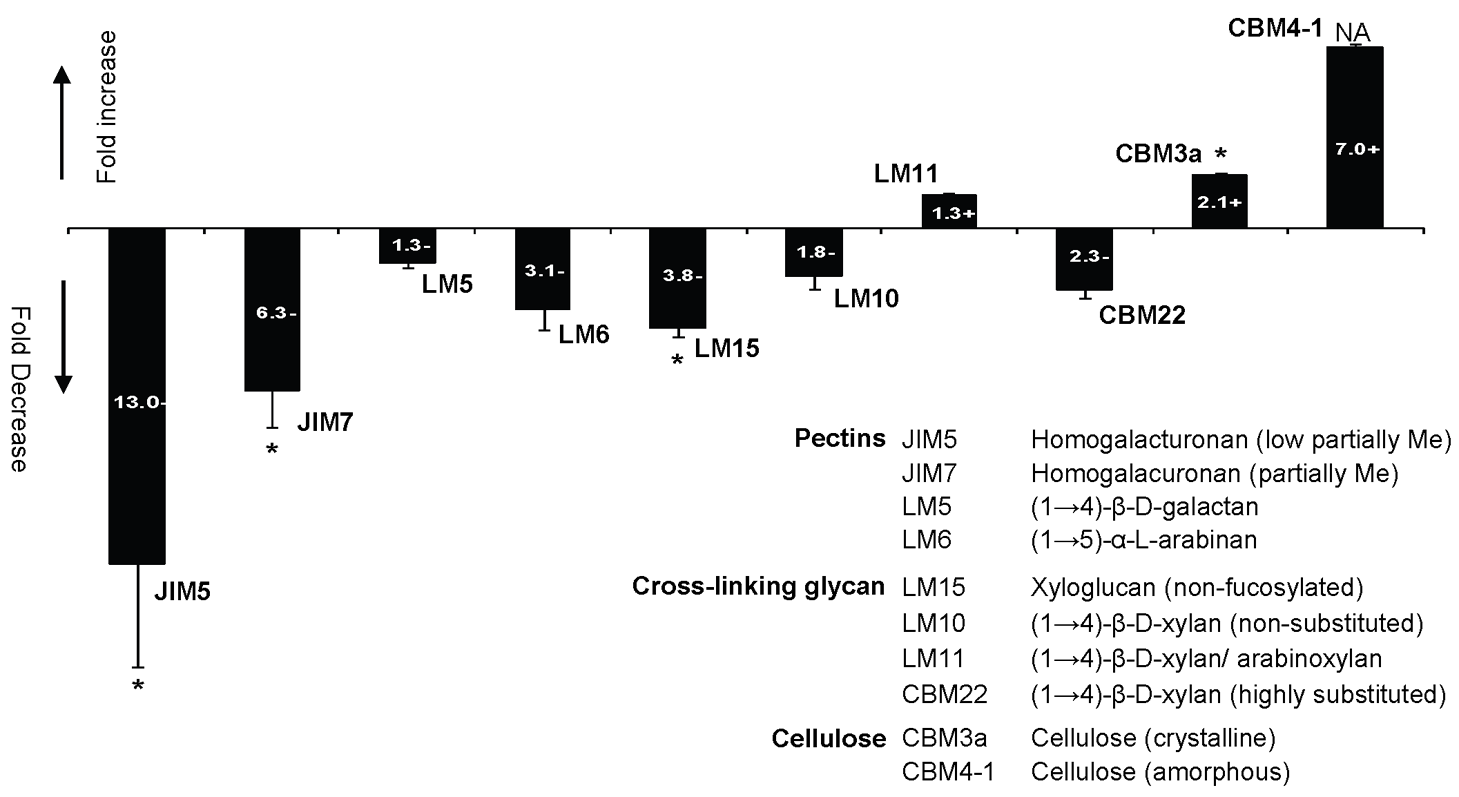

Supplement: Figure S1 — Fold changes (decreases or increases) in polysaccharide occurrence. Numerical values represent the degree of change in polysaccharide occurrence between the leaf material input to the fungus garden and the debris output from the fungus garden. Individual mAbs and CBMs and their corresponding polysaccharide epitopes are listed in the figure. * indicate that fold-changes were significantly different from 1 (ANOVA, p < 0.05). NA indicates that statistical analysis could not be performed on CBM4-1 as this epitope was only available for analysis from two colonies. Error bars represent standard error (±SE). (TIF) [file pone.0017506.s004.tif]

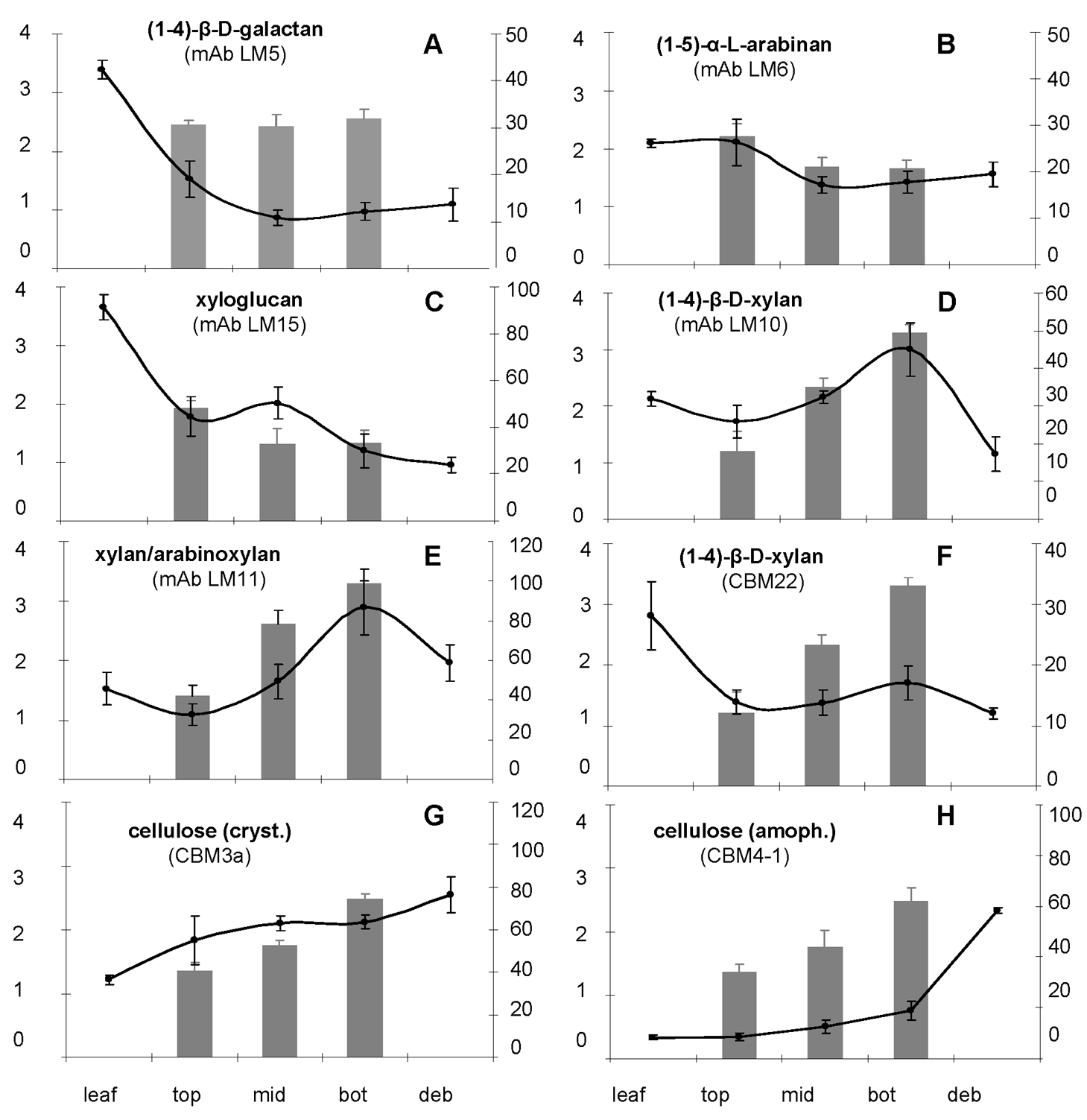

Supplement: Figure S2 — Enzyme activity in fungus gardens of Acromyrmex echinatior . Bar graphs (A-H, left y-axis) indicate enzyme activity as halo area (cm2) in the three different sampling locations of the fungus garden as determined by AZCL-polysaccharide plate assays. Line graphs (A-H, right y-axis) indicate relative abundances of the corresponding polysaccharides as determined by CoMPP analysis (the same data as in Figure 3 but not adjusted to become a fraction of 100). Enzyme substrates and the corresponding mAbs or CBMs used in the analysis are indicated for each graph (see also Table S1). Enzyme data for (1-4)-β-D-xylan are shown twice (D and F) as both mAb LM10 and CBM22 detect this substrate. Error bars represent standard errors across four colonies measured. (TIF) [file pone.0017506.s005.tif]
